# Tracing early stages of species differentiation: Ecological, morphological and genetic divergence of Galápagos sea lion populations

**DOI:** 10.1186/1471-2148-8-150

**Published:** 2008-05-16

**Authors:** Jochen BW Wolf, Chris Harrod, Sylvia Brunner, Sandie Salazar, Fritz Trillmich, Diethard Tautz

**Affiliations:** 1 Institute for Genetics, Evolutionary Genetics, University of Köln, 50674 Köln, Germany; 2 Max-Planck Institute for Evolutionary Biology, Evolutionary Genetics, 24306 Plön, Germany; 3 Ecology and Evolutionary Biology, School of Biological Sciences, Queen's University Belfast, 97 Lisburn Road, BT9 7BL, UK; 4 Museum of the North, University Alaska, 907 Yukon Drive, Fairbanks, AK 99775, USA; 5 Estación Científica Charles Darwin, Puerto Ayora, Galápagos, Ecuador; 6 Department of Animal Behaviour, University of Bielefeld, PO Box 10 01 31, 33501 Bielefeld, Germany

## Abstract

**Background:**

Oceans are high gene flow environments that are traditionally believed to hamper the build-up of genetic divergence. Despite this, divergence appears to occur occasionally at surprisingly small scales. The Galápagos archipelago provides an ideal opportunity to examine the evolutionary processes of local divergence in an isolated marine environment. Galápagos sea lions (*Zalophus wollebaeki*) are top predators in this unique setting and have an essentially unlimited dispersal capacity across the entire species range. In theory, this should oppose any genetic differentiation.

**Results:**

We find significant ecological, morphological and genetic divergence between the western colonies and colonies from the central region of the archipelago that are exposed to different ecological conditions. Stable isotope analyses indicate that western animals use different food sources than those from the central area. This is likely due to niche partitioning with the second Galápagos eared seal species, the Galápagos fur seal (*Arctocephalus galapagoensis*) that exclusively dwells in the west. Stable isotope patterns correlate with significant differences in foraging-related skull morphology. Analyses of mitochondrial sequences as well as microsatellites reveal signs of initial genetic differentiation.

**Conclusion:**

Our results suggest a key role of intra- as well as inter-specific niche segregation in the evolution of genetic structure among populations of a highly mobile species under conditions of free movement. Given the monophyletic arrival of the sea lions on the archipelago, our study challenges the view that geographical barriers are strictly needed for the build-up of genetic divergence. The study further raises the interesting prospect that in social, colonially breeding mammals additional forces, such as social structure or feeding traditions, might bear on the genetic partitioning of populations.

## Background

The relative role of ecologically mediated divergence in speciation processes is still under debate [1]. Theory predicts that barriers to gene flow can evolve as a result of ecologically-based divergent selection and need not necessarily be associated with separation imposed by geographic barriers [2-5]. Recent empirical evidence makes it increasingly clear that ecological factors can indeed drive speciation processes [6-9]. Traditionally, top-down phylogenetic analyses, where the relevant divergence processes are inferred retrospectively long after the putative split has occurred have often been invoked to address this question. While this is clearly a powerful approach to reveal evolutionary trajectories, it is by its very nature restricted to retrospective inferences and can thereby only speculate about the ecological conditions under which the speciation process was initiated. It is hence necessary to identify cases where the first steps of divergence appear, even if one can not definitely know whether it will eventually end with a true species separation [10-13]. Studying ongoing differentiation processes in small-scale situations with unlimited dispersal opportunities is therefore crucial to investigate the mechanisms driving adaptive divergence.

Marine environments provide excellent study cases, as they typically allow broad dispersal in mobile taxa and, compared to terrestrial habitats, offer low travel costs [14]. Still, within geographic ranges of several thousand kilometres genetic isolation by distance is expected and has been observed even for highly mobile marine predators [15]. However, it is a challenge to track evolutionary divergence processes at a smaller spatial scale. The few that have ventured on this undertaking have produced interesting results ranging from a role of gamete recognition molecules [16] to a role of socially mediated information [17]. We here present a system that allows examination of micro-evolutionary processes in an isolated, small-scale marine environment for a highly mobile top predator.

**Figure 1:**
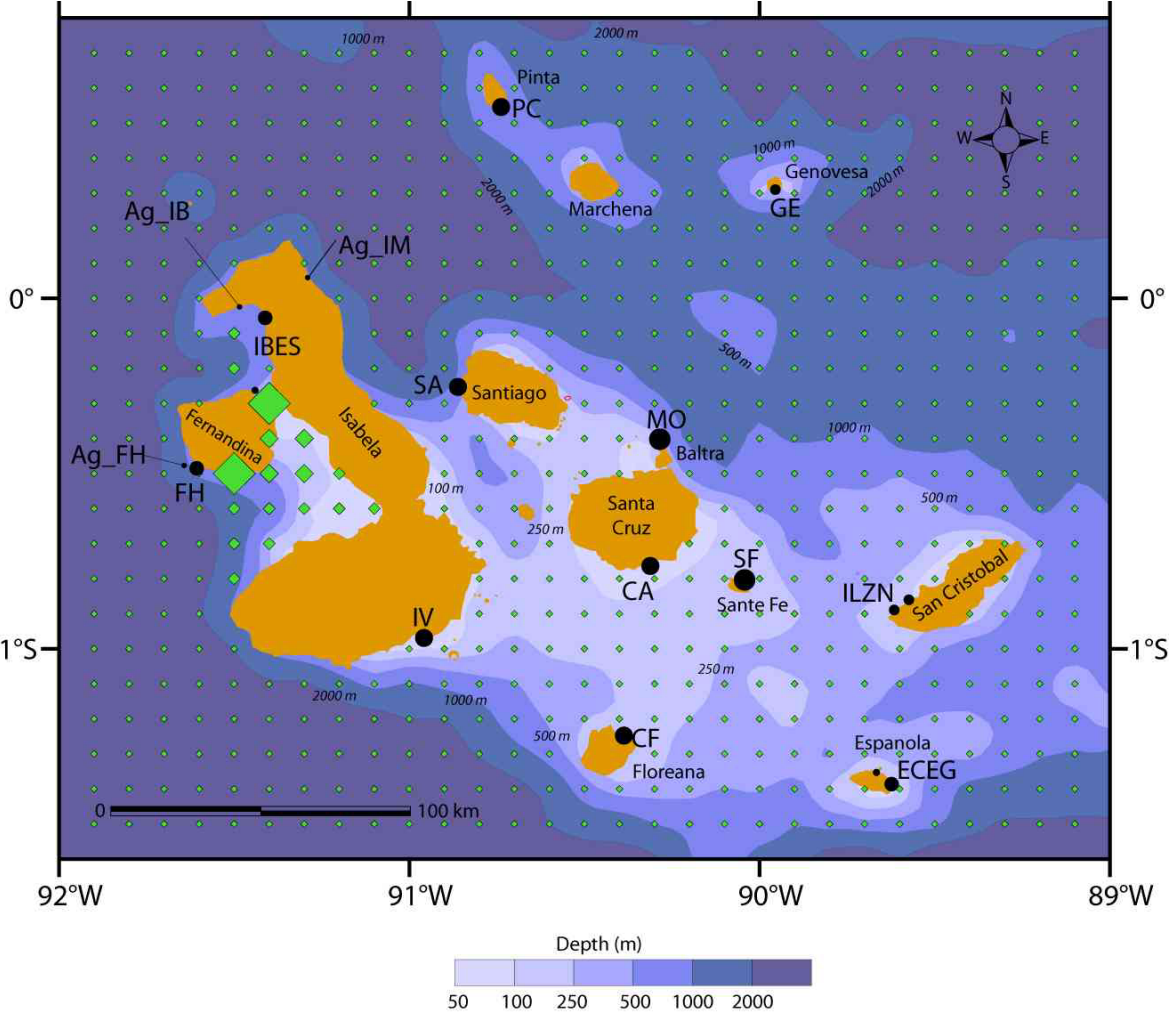
Map of Galápagos sea lion (*Zalophus wollebaeki*) rookeries sampled across the Galápagos archipelago. Dot size reflects the number of sampled individuals. Sampling locations are generally labelled by a two-letter code. Where rookeries have been pooled due to sample size limitations they are encoded with four letters. Rookeries of the Galápagos fur seal (*Arctocephalus galapagoensis*) are indicated by the prefix *Ag*. Diamonds symbolize the average chlorophyll a concentration from 1998–2007 SeaWiFS satellite data indicative for the nutrient level of a given location (symbol size scales linearly with chl a concentration ranging from 0.216–6.339 mg/m^3^). For details of sampling locations and sample sizes for mtDNA marker, 22 nuclear microsatellites markers and stable isotope analysis see Table 1.

The Galápagos sea lion (*Zalophus wollebaeki*) is endemic to the archipelago and genetically distinct from its nearest relatives [18]. Thus, any differentiation that can be traced within the archipelago must be genuine and will not due to an allopatric past with following reinvasion. Its marine ecosystem is divided into two distinct habitats (Fig. 1, Table 1): Fernandina and the west-coast of Isabela differ from its east-coast and all remaining islands in bathymetry, water temperature and nutrient content [19]. While waters on the central plateau are shallow ('Centre' hereafter), the sea west of Fernandina drops rapidly to depths of several kilometres. Central waters are relatively warm and low in nutrients; the 'West', in contrast, is influenced strongly by the cold upwelling waters of the Cromwell current. Such variation in physical properties between the areas results in considerable ecological differences. Primary production is markedly higher in the west, and is particularly pronounced in the area east of Fernandina, where iron concentrations are highest [20]. The distribution of animals dependent on aquatic resources mirrors the ecological differences between these contrasting habitats. Viable populations of endemic sea birds as well as colonies of the second Galápagos seal species, the Galápagos fur seal (*Arctocephalus galapagoensis*), are essentially confined to the more productive western habitat [21,22]. In contrast, the distribution of the Galápagos sea lion includes both habitats. This results in a rather special situation, where both intra- as well as inter-specific niche differentiation between the two seal species could act as ecological sources of selective divergence. It poses the question, whether such environmental contrasts can translate into genetic divergence in a species with a basically unlimited dispersal capacity across its entire range.

**Table 1:** Sampling locations and sample sizes

Taxa	Island (code on map)	Coordinates	Number of samples			Differentiation scenario: geological/ecological
		
			≥ 625 bp mtDNA	Amplifying ≥ 21 microsatellite loci	Stable isotope analysis	
*Zalophus wollebaeki* (Galápagos sea lion)	Caamaño (CA)	0°46'58''S, 90°17'42''W	27	30	10	group 1/Centre
	Floreana (CF)	1°14'16''S, 90°23'16''W	30	29	11	group 1/Centre
	Mosquera (MO)	0°24'58''S, 90°16'42''W	40	40	10	group 1/Centre
	Santiago (SA)	0°14'18''S, 90°52'25''W	29	30	10	group 1/Centre
	Santa Fé (SF)	0°48'18''S, 90°02'25''W	35	39	10	group1/Centre
	Española * (ECEG)	1°22'07''S, 89°38'32''W	29	28	19	group 2/Centre
	San Cristobal* (ILZN)	0°52'30''S, 89°36'00''W	23	47	--	group 2/Centre
	Pinta (PC)	0°32'10''N, 90°44'20''W	30	30	10	group 3/Centre
	Genovesa (GE)	0°18'16''N, 89°57'16''W	13	14	14	group 3/Centre
	Isabela (IV)	0°57'58''S, 90°57'42''W	30	30	--	group 4/Centre
	Fernandina (FH)	0°28'18''S, 91°36'25''W	23	23	22	group 4/West
	Isabela *(IBES)	0°09'44''S, 91°25'25''W	27	27	24	group 4/West

*Arctocephalus galapagoensis* (Galápagos fur seal)	Fernandina (Ag_FH)	0°28'11''S, 91°37'38'' W	--	--	30	
	Isabela Banks Bay (Ag_IB)	0°01'09''S, 91°29'52''W	--	--	30	
	Isabela Marshal Bay (Ag_IM)	0°03'58''N, 91°17'12''W	--	--	30	

*Zalophus californianus* (Californian sea lion)	Año Nuevo Island	37°06'N, 122°19'W	--	14	--	
	Moss Landing Beach	36°47'N, 121°47'W	--	2	--	

TOTAL			336(GSL)	367(GSL)	140(GSL)	
				11(CSL)	90 (GFS)	

Colony locations, number of samples in final analyses and differentiation scenario used for estimation of hierarchical population differentiation for the Galapagos sea lion (GSL), the Californian sea lion (CSL) and the Galapagos fur seal (GFS). Locations that were pooled due to sample size limitations are labelled with an asterisk.

## Results

### Ecological divergence

**Figure 2:**
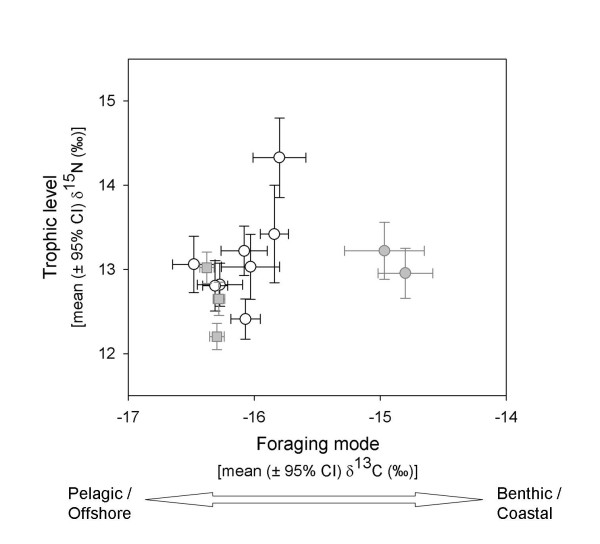
Isotopic biplot showing mean (± 95% CI) of δ^13^C and δ^15^N values from juvenile Galápagos sea lions (circles) and Galápagos fur seals (square) collected in different rookeries across the Galápagos Islands. The corresponding habitat of each rookery is indicated by colour (white = Centre, grey = West).

The Galápagos sea lion and the Galápagos fur seal were sampled extensively across their distribution ranges. Stable isotope analysis was used to provide insight into foraging ecology. δ^15^N values reflect differences in trophic levels of prey items, whereas δ^13^C values indicate foraging mode [pelagic or benthic: see e.g. [23,24]]. Although both sea lions and fur seals are characterized generally as pelagic foragers, we see differences in stable isotope signature values between syntopic populations of these species. While mean δ^13^C values overlap between fur seals and central sea lion colonies, values from western sea lion colonies are displaced significantly (Fig. 2). Quadratic discriminant function analysis underpins the difference between sea lion colonies of different habitats (Wilk's λ = 0.336, F_3,136 _= 89.6, p < 0.001). The overall jacknifed classification success between the different sea lion populations was as high as 95% (Table 2A), indicating a clear isotopic differentiation between the two habitats.

**Table 2:** Summary statistics of discriminant function analysis

	a priori classification	West	Centre	Classification success [%]
A) Stable isotopes	West	**41 (40)**	5 (6)	89 (87)
	Centre	2 (2)	**92 (92)**	98 (98)
	Total	43 (42)	97 (98)	95 (94)
				
B) Morphometry	West	**9 (6)**	0 (3)	100 (67)
	Centre	4 (7)	**23 (20)**	85 (74)
	Total	13 (13)	23 (23)	89 (72)

Classification success and jacknifed classification success (in brackets) of the discriminant function analysis using A) stable isotope signatures and B) multiple morphometric measurements as the predictor variable. Classification success describes the predictive accuracy with which an individual is correctly associated with any of the classes of interest that were defined *a priori*. Correctly classified individuals are shown in bold.

We further tested for homogeneity of variance in the isotopic signal that can be indicative of niche width differences [25]. For two pairs of directly adjacent populations of sea lions and fur seals (IBES/Ag_IB and FH/Ag_FH, see Fig. 1) variances in δ^13^C values are larger in sea lions (IBES/Ag_IB: F_23,29 _= 34.90, p < 0.001, FH/AgFH: F_21,29 _= 9.92, p < 0.001), while differences in δ^15^N values are statistically non-significant after correcting for multiple testing (IBES/Ag_IB: F_23,29 _= 2.30, p = 0.04; FH/AgFH: F_21,29 _= 2.57, p = 0.02).

### Morphological divergence

**Figure 3:**
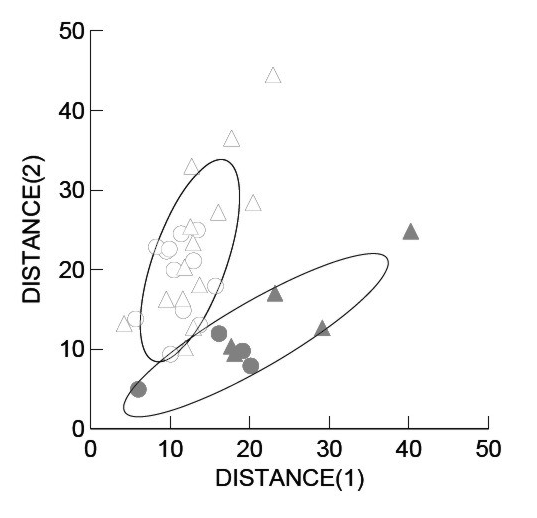
Mahalanobis distances of several foraging-related skull morphometric measurements with 95% confidence ellipses for female (circles) and male (triangles) adult Galápagos sea lions of either habitat (white = Centre, grey = West).

Analyses of skull features also show a differentiation between the western and central colonies which may be related to different foraging strategies (Fig. 3). Skulls from western habitats are generally smaller, yet more robust, than those from the central group. Mean condylobasal length of adults are larger in central specimens than in those from the western habitat (see Additional file 1). Variables that contribute most to inter-habitat variation are: breadth of skull at preorbital processes, palatal notch – incisors, length of upper postcanine row, rostral width, gnathion – posterior border of preorbital process and palatal breadth. Breadth of skull at preorbital processes, auditory breadth, and palatal breadth are greater in western specimens than in central ones, both in *mm *and as a percentage of condylobasal length. Although absolute rostral width values are similar in specimens from both habitats, it appears greater in western specimens than in central individuals when considered as a percentage of condylobasal length. Rostral length appears shorter in western specimens than in central individuals, again indicating a shorter, yet more robust, skull in western individuals. Discriminant function analysis shows that specimens of the two habitats (west n = 27; central n = 9) are clearly separated from one another (Wilk's λ = 0.360, F_13,22 _= 3.013, p < 0.01). The jack-knifed classification matrix successfully classifies 72% of specimens to the right colony (Table 2B).

### Genetic divergence

**Figure 4:**
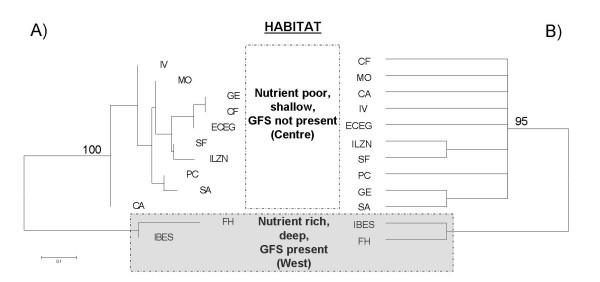
A) Neighbour-joining tree of mitochondrial DNA showing genetic relationships among rookeries of Galápagos sea lions. Genetic distances between rookeries are based on corrected mean pair-wise sequence comparisons of the mitochondrial control region. B) 50 percent Neighbour-joining bootstrap consensus tree based on Goodman's Rst at the rookery level for 22 microsatellite loci. Bootstrap support values (5000 replicates) are shown above the nodes. Abbreviations: GFS = Galápagos fur seal, letter codes represent sampled populations (see Fig. 1)

Analysis of mitochondrial sequences supports the pattern of ecological and morphological divergence. Among the three models tested (see Methods and Table 1) genetic variation can be attributed almost exclusively to habitat structure (AMOVA: Φ_st _= 0.224, p < 0.001), whereas the other models of hierarchical population structure explain far less variation (colony pair-wise: Φ_st _= 0.086, geology: Φ_st _= 0.097, p_both _< 0.001). After correcting for habitat the variance component of the colony-pair wise comparison gets non-significant and explains only 1.2% of the overall variance. A neighbour-joining tree based on mean corrected pair-wise distance between colonies further confirms the split (Fig. 4A).

Analysis of genetic differentiation at the level of microsatellites and the individual colonies using Goodman's standardized Rst as the pair-wise distance suggests the same habitat-related pattern (Fig. 4B). This split is corroborated by global estimates of traditional fixation indices (Rst = 0.020; Gst' = 0.012, θ = 0.012: bootstrapped CI_99% _= 0.005–0.021; G-statistic: p < 0.001).

**Figure 5:**
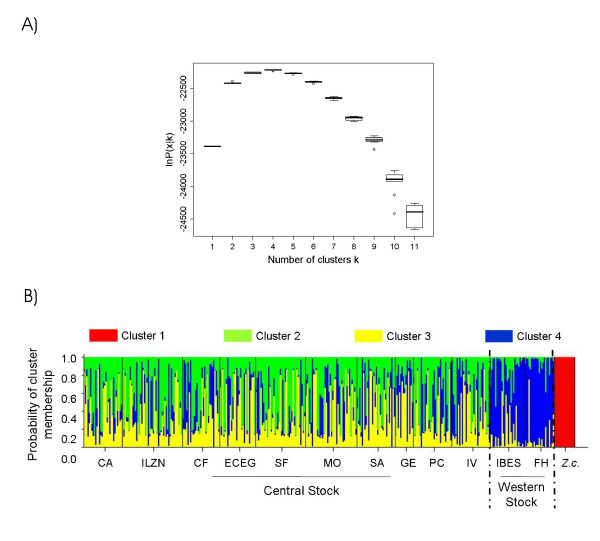
A) Results from ten independent runs of STRUCTURE 2.1 [70] for each hypothesized number *k *of genetically meaningful clusters using 16 Californian and 367 Galápagos sea lions. Posterior probabilities *ln P(x|k) *indicate which number of populations are most likely to explain the genotypic data. B) Barplot of membership probabilities for the scenario of population subdivision that was best supported by the data (*k *= 4). Each individual is represented by a stacked bar that can be partitioned into a maximum of four differently shaded segments, each standing for a genetic cluster. The probability of cluster membership is portrayed by relative segment length for each individual. Colonies of origin and genetic stocks are given below, the Californian sea lion (Z.c.) is included as the outgroup (see Methods).

As a further test for nuclear genome differentiation, we used a Bayesian assignment approach. This has the advantage that inferences are made in the absence of any *a priori *assumptions inherent in hierarchical frequentist approaches. Overall, four clusters best explain the genetic structure in the dataset (Fig. 5A). As expected, the Californian sea lion which was used as an outgroup (see Methods) forms a distinct cluster of its own (Fig. 5B, see Additional file 2). Within the Galápagos archipelago the existence of three genetic clusters is suggested. Assigning the individuals to clusters in which membership coefficients are greatest shows that one cluster (cluster 4) corresponds to the western colonies with 85% of the individuals assigned correctly (Fig. 5B, see Additional file 2). Membership of the remaining two central clusters is evenly distributed across the central populations and no geographical correlate thereof can be deduced (Additional file 2). When these clusters are combined, 78% of the individuals are assigned correctly to their origin in the centre of the archipelago with a high mean membership coefficient of 0.76 ± 0.02SE.

### Isolation by distance

**Figure 6:**
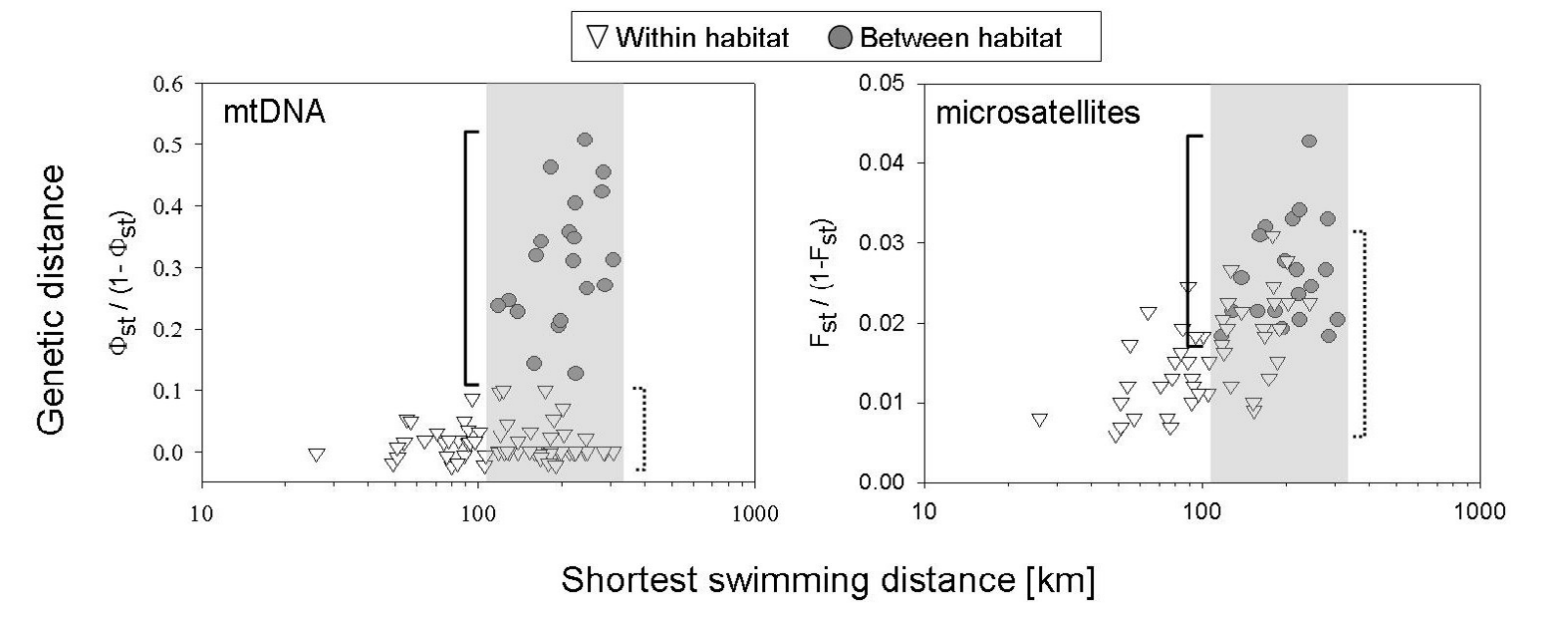
Relationship of geographic distance (logarithm of shortest swimming distance) and genetic distance of the mitochondrial (mtDNA) and nuclear marker (microsatellites). Triangles symbolize pair-wise comparisons between rookeries that share the same habitat. Filled circles stand for comparisons across habitats. The shaded area indicates the range of geographic distances that is characteristic for both intra- and inter-habitat specific pairwise comparisons. Dotted brackets visualise the value range of genetic distances from within habitat comparisons, solid brackets from between habitat comparisons.

We further explored the possibility that geographic distance contributes to genetic differentiation. Indeed, microsatellites as well as mitochondrial DNA data suggest isolation by distance (Mantel test mtDNA: R^2 ^= 0.37; nDNA: R^2 ^= 0.46; p_both _< 0.001). However, in the case of mitochondrial data, the correlation only reflects the habitat split (West versus Centre; Fig. 6). After partialling out the effect of habitat the evidence for isolation by distance disappears (partial Mantel test: R^2 ^= 0.04, p = 0.25). For microsatellite data, pair-wise comparisons of colonies from the same habitat still follow a statistically significant, but weaker, isolation by distance pattern (partial Mantel test: R^2 ^= 0.25, p < 0.001). The overall degree of scatter in the genetic distance measure significantly increases with geographic distance indicating equilibrium between gene flow and drift in a stepping stone model of migration (partial Mantel: R^2 ^= 0.56, p < 0.001) [compare e.g. [26]].

Another noteworthy difference between the isolation by distance pattern of mtDNA and nDNA relates to the variance of the genetic distance measure. For comparable geographic distances the variance in Fst between populations within habitats (dotted brace in Fig. 6) and between habitats (solid brace in Fig. 6) is of similar size for nDNA (F_19,45 _= 1.14). For mtDNA the variance of Φ_st _for inter-habitat comparisons is four times as large as the variance of intra-habitat comparisons (F_19,45 _= 4.12).

## Discussion

Using ecological, morphological and molecular indicators, we find a clear structure between western and central Galápagos sea lion colonies, even though these are extremely mobile predators and breeding dispersal is potentially unrestricted across the entire species range. The mobility potential is well exemplified by the Californian sister species of the Galápagos sea lion [18] that can easily travel several hundred kilometres during foraging routines [27]. Similarly, for the Galápagos sea lion satellite telemetry data show that the scale of ecological and genetic divergence lies well within the geographic range of daily foraging trips [28]. In other marine mammals of similar mobility [15] including species of seals [29] a comparable degree of genetic differentiation is usually found only at geographic scales that are about 10-fold larger. This is not surprising, as high mobility usually translates into strong gene flow. In elephant seals for example, a single male can successfully father 19 offspring 8000 km from its natal rookery [30]. This calls for an explanation beyond mere distance effects in the Galápagos sea lion, where homogenizing effects of even rare dispersal events would equally be expected as in other polygynous animals. In the following, we discuss the possible factors that might play a role in this differentiation.

### A role of ecology

Using the results of stable isotope analysis as a proxy for maternal trophic ecology we find that individuals of the Galápagos sea lion cluster according to their natal habitat. Colonies in the central habitat are characterized by pelagic shelf feeding, a foraging strategy that is also typical for the closely-related Californian sister species. Conversely, colonies found adjacent to the deep, nutrient rich habitat in the west show an atypical benthic signature. This difference in isotopic signatures between western and central populations could simply reflect differences in food-web-wide basal isotopes. It is however intriguing that the fur seal, which overlaps with the sea lion in this habitat, shows the typical pelagic δ^13^C values of eastern sea lions. This counters the idea that differences in basal isotopes of the foraging location alone account for the observed difference in the sea lions. It is rather indicative of resource partitioning, potentially via character displacement in this area where competition for a joint resource leads to specialization of at least one of the competing species. Grant and Grant [31] have shown in two species of Galápagos finch that such character displacement can occur rapidly. As stable isotope values integrate maternal foraging strategies over several months (see Methods), the measured effect could develop within even a single generation. On the other hand, the changes in skull features are likely to require longer periods of directional selection, suggesting that the differences in foraging strategies are established and stable in the respective populations.

It is clear that the data presented here can only be a first hint towards such character displacement and need to be substantiated by several independent lines of evidence that go beyond the scope of this study [32]. Nonetheless, other sources of information on Galápagos fur seals and sea lions indirectly corroborate the interpretation of our data as being indicative of niche segregation. Fur seals forage at night, western sea lions exclusively during the day [33], whereas central sea lions show no apparent temporal pattern [unpublished data from long-term study on a central rookery [34]]. Scat analyses revealed that, in contrast with central sea lions that are using a broad prey spectrum [35], trophic niches of fur seals and western sea lions are highly specialised and show little overlap [36]. Furthermore, sea lions from the different habitats seem to diverge in diving behaviour [28,37]; hence, dissimilar ecological conditions within the archipelago and the competition with fur seals in one habitat appear to evoke habitat specialisation in the sea lion.

### A role of natal habitat preference induction and social behaviour

To develop levels of genetic differentiation that reflect the ecological differentiation between different populations of Galápagos sea lions, some form of pre-zygotic isolation is required. Habitat choice would be one such mechanism and could constitute a non-genetic means of assortment. There is convincing theoretical and empirical evidence that habitat preferences can be based solely on learning [4,38]. For instance, early learning can lead to a lifetime shift in feeding niche, even across species [39]. Natal habitat preference induction is particularly likely to evolve in species with long lasting social bonds between adults and young. The discussion regarding the role of socially mediated feeding styles of killer whales as the primary source of genetic differentiation is a prominent example [17]. Likewise, genetic divergence between transient and resident wolf populations links to different foraging strategies and suggests a similar explanation [40].

Galápagos sea lions are highly social animals, whose offspring are dependent on their mother for one to three years [41]. They are likely to have the same long-term memory [42] and high cognitive abilities as their Californian sister species [43]. The idea of socially mediated habitat learning thus seems not far fetched and is partly supported by telemetry data on female Galápagos sea lions. None of the surveyed females ever crossed the habitat border in any of the recorded foraging trips, although it lay well within their mobility capacity [28]. In addition to the "ecological" habitat the social environment may contribute to reducing gene flow. In contrast to other species that only join for reproduction, Galápagos sea lions maintain haul-out sites year round. In such a situation, reproductive success is likely to be affected by long-term interactions with others [34] and predictability of the social environment is of prime importance [44-46]. Thus, learned habitat preference induction – be it ecological or social – may well contribute to pre-zygotic isolation.

The observed isolation by distance pattern strengthens this idea. The mitochondrial marker reflecting matrilineal inheritance shows no relationship between genetic and geographic distance after habitat identity is removed as a factor. Thus, within one habitat, gene flow seems relatively unrestricted and genetic variants can spread across the entire central region. This homogenizing effect of gene flow that is witnessed by the absence of isolation by distance and the low variance of intra-habitat comparisons suggests that site fidelity alone [44] is not strong enough in this species to create significant population structure as reported in other otariid seals [47]. Hence, environmental differences seem to be key to the understanding of genetic divergence. This is corroborated by the fact that the variance of genetic distance between rookeries of different habitats is much larger than among rookeries of the same habitat indicating that drift across habitats is strong relative to gene flow.

For microsatellites the isolation by distance pattern is in line with a stepping stone model of a regional equilibrium with gene flow and drift [compare [26]]. This clear difference from the mitochondrial pattern is not easy to explain and may partly be due to the fact that differentiation of the two markers differs by an order of magnitude. It may further be due to the four times smaller effective population size of the mitochondrial marker or differences in mutational dynamics between the two marker systems. The most compelling explanation might lie in the large difference in information content of the two markers. While mitochondrial results are based on a short stretch of sequence data in one locus, the results of nuclear DNA stem from 22 independent highly variable microsatellite loci. The information for the mtDNA may thus simply not suffice to pick up the isolation by distance pattern between populations sharing the same habitat.

Another factor bearing on the isolation by distance patterns could also be sex specific migration behaviour. The nuclear pattern suggests that males are more likely to cross occasionally the habitat boundaries, but would on the other hand show high site fidelity even within the respective habitats, together with the females. While female site fidelity is characteristic for most mammalian species [48], short range dispersal in males is less common. Why then would males restrict their dispersal to an area that is even smaller than their daily putative foraging range? In contrast to other species that only join for reproduction, the sea lion adult males are known to reside for years [Pörschmann et al. in prep, [49]]. In such a situation reproductive success is likely to be affected by long-term interactions with others [34] and predictability of the social environment is of prime importance [44,45]. For males, in particular, long-term social dominance hierarchies, social queuing and 'dear enemy relationships' are essential for territorial success [50-52]. The fact that males of the Antarctic fur seal (*Arctocephalus gazella*) return to locations at a scale less their own body length year after year [53] and males that are able to establish territories several years in a row increase reproductive success [54] can be interpreted along these lines.

### A role of selection against immigrants

Apart from natal-induced habitat preference, an alternative mechanism that may contribute to pre-zygotic isolation was described by Hendry [55]. In a modelling approach he proposed that selection against migrants themselves can contribute substantially to ecologically dependent reproductive isolation. Nosil et al. [56] even suggested that this mechanism plays a critical role in ecological modes of speciation. Given the difference in ecology and the apparent behavioural and morphological adaptations in the West, we might expect that immigrant sea lions from the central area would have problems to compete successfully with resident animals. Thus, once ecological differentiation has been initiated, this factor would stabilize any genetic divergence.

### A role of geography and geology

The geology of the Galápagos can be described as a combination of concentrated volcanic activity at the archipelago's western rim (hotspot) and lithospheric motion that carries the emerging volcanoes off in a north-eastern direction. This results in a shallow submarine platform with steep abysses at its western and southern side that gently slopes to the north-east where it joins the intersection of two major tectonic plates [57]. These geological processes lead to an almost linear island age structure across the archipelago: easternmost islands are oldest (San Cristobal, Española ~3 mya), westernmost islands are youngest (Fernandina: ca. 0.08 mya [58]). Assuming comparable oceanographic conditions to those of today, we would expect similar habitat differences across the archipelago over geological times. Without any geographic barriers, the cold upwelling western waters would mix with warmer waters in the east, and ecological differences would most likely resemble an environmental gradient. It has been shown that such environmental gradients can trigger genetic divergence into two discrete states in models of sympatric divergence [5]. The emergence of Isabela in the west would have further accentuated this. The large northern and southern volcanoes of Isabela emerged about 0.2–0.4 mya ago [59] and probably joined only within the past few thousand years (D. Geist personal communication).

## Conclusion

Our data show evidence for intra-specific divergence of the Galápagos sea lion at ecological, morphological and genetic levels, which may potentially lead to the emergence of a new species over time. Our analysis shows that a multitude of factors may play a role in ecological divergence, including some behavioural conditions that are specific to the system. In particular, the data constitute an example where substantial effects of a competitor species on intra-specific evolutionary processes appears likely [31,32]. Geographic isolation, on the other hand, seems to play only a small role. Thus, our results are in line with an increasing number of studies that suggest that the current dominance of allopatric and parapatric speciation concepts in evolutionary theory may be in part an artefact of studying speciation patterns at levels where the processes have long been completed. The study highlights that divergence processes are likely to be based on a variety of factors, and that little will be gained by exclusively adhering to a controversial debate about geographic speciation scenarios [7].

## Methods

### Tissue sample collection and DNA extraction

A total of 376 tissue samples were collected from the inter-digital membrane of the hind flippers from newborn individuals of the Galápagos sea lion and the Galápagos fur seal at their natal colonies. Sampling locations were spread uniformly across the Galápagos archipelago excepting the northernmost islands of Darwin and Wolf (Fig. 1, Table 1). Adjacent colonies with low individual sample sizes were pooled, their geographic position averaged and subsequently treated as one entity (indicated by four letter codes in Fig. 1). Samples of the Californian sea lion were supplied from locations central to the taxon's range containing adults (n = 5) as well as sub-adults (n = 11) (Table 1).

### Stable isotope analysis

Skin samples for stable isotope analysis were taken from a total of 140 the Galápagos sea lion pups and from 90 Galápagos fur seal pups (Table 1) that were about three months old. This is an age where pups are nutritionally fully dependent of their mothers [41]. The stable isotope signature therefore exclusively represents maternal foraging strategies. Skin samples were oven dried at 65°C for 24 h. Samples were pulverised, weighed (ca. 0.55 mg) and loaded into tin cups prior to analysis of carbon (δ^13^C) and nitrogen (δ^15^N) stable isotope ratios [for analytical details see [60]]. Analytical precision was < 0.1‰ (δ^13^C) and < 0.3‰ (δ^15^N).

In order to examine whether isotopic and elemental variation in skin samples represented a viable means to differentiate the different genetic stocks and species, we ran a discriminant function analysis using δ^13^C, δ^15^N and C:N values as predictors of stock/species following Harrod et al. [61]. We used a quadratic discriminant function as our sample size differed between groups and because of heterogeneity of variance in some variables.

### Morphometric analysis

A total of 43 skulls of the Galápagos sea lion held at several natural history museums and institutions (see Additional file 3) were measured for the following 13 variables using Mitotoyo digital calipers (accuracy ± 0.01 mm): condylobasal length, breadth of preorbital processes, interorbital constriction, palatal notch – incisors, length of upper postcanine row, rostral width, gnathion – posterior of maxilla (palatal), breadth of zygomatic root of maxilla, zygomatic breadth, basion – zygomatic root (anterior), auditory breadth, gnathion – posterior border of preorbital process, palatal breadth at postcanine five. All skulls were used for univariate statistics; thirty-six of these (those with no missing variables) were used for discriminant analyses. Only fully grown adult specimens with suture indices of > 23 for males and > 18 for females were included in the analyses [62]. Raw data were initially standardized to *z*-scores so that each variable had equal weighting. Specimens were grouped according to the habitat where they were collected. Note that this leads to a conservative classification estimate, since skull samples may include occasional visitors that originate from other habitats. Discriminant function analysis using SYSTAT 11 was applied to examine relationships between individuals from the different habitats. Multivariate ANOVA (MANOVA) was followed by either two-group or multi-group discriminant function analysis. The MANOVA was applied initially to test whether group centroids for specimens were significantly different. Mahalanobis distances of individuals from the mean centroid were plotted for each habitat, against discriminant axes I and II. When sexes were analyzed separately, both males and females showed similar Mahalanobis distances. Due to low numbers of individuals from the western habitat (males = 5, females = 4) sexes were pooled to provide greater resolution of results.

### Mitochondrial DNA: laboratory procedures and data analysis

After extraction of genomic DNA, the mitochondrial control region was amplified by use of PCR with taxon-specific modifications of highly conserved primers located in the tRNA^thr/pro ^and the tRNA^phe ^region, purified by ultrafiltration and sequenced on an ABI 3730 sequencer [18]. Quality ascertainment and sequence alignment were conducted in SEQMAN™ version 6.1. (DNAStar Inc.). Individuals with less than 625 bp of reliably identified sequence were excluded from the analysis leaving a total of 336 individuals. From these, 29 haplotypes can be distinguished. If alignment gaps are included as a fifth character the number of haplotypes rises to 36. Sequences for all individuals and the haplotype alignment are deposited as alignment ALIGN_001234 in the EMBL-Align database that can be accessed by the EBI sequence retrieval system [63].

Φst was inferred by AMOVA as implemented in ARLEQUIN 3.10. [64] and used as an estimator of hierarchical population differentiation of the mitochondrial genome. We compared three scenarios (see Table 1): comparisons among colonies a) without any further hierarchical level, b) grouping colonies by island geology following Rassmann et al. [65] c) grouping colonies by habitat type. Genetic distances were based on the K80 nucleotide substitution model, which is closest to the substitution model suggested by Wolf *et al*. [18]. Qualitatively, results were unaffected by whether alignment gaps were or were not included in the analysis.

### Nuclear DNA: microsatellite genotyping and data analyses

Genomic DNA was genotyped for a total of 367 Galápagos sea lion and 16 Californian sea lions at 22 microsatellite loci [for further details see [18,66,67]]. Population structure was inferred using the program STRUCTURE [68] including the Californian sea lion in the analysis, as otherwise the MCMC would not converge. Evanno et al. [69] proposed an ad hoc statistic, Δk, to detect the number of clusters that best fit the dataset. We did not adhere to this procedure for two reasons: firstly, it is not suited to resolve less than three clusters and secondly, it may lead to unreliable results, as the calculation of Δk includes several chains that may have not converged. We therefore followed the original method by Pritchard [70], namely to run several chains (10) and for each value of k select the MCMC run with the smallest value of -log(Pr(x|k)). Conventional Fst [71] and Rst estimates [72] were used to estimate the degree of genetic differentiation between the inferred populations using FSTAT 2.9.3.2. [73]. The G statistic proposed by Goudet et al. [74] was taken for statistical inference on global population differentiation. Bootstrapped pair-wise Rst_(Goodman) _distances were obtained from the software MICROSAT 1.5d [75] and used for cluster-based tree reconstruction in the PHYLIP module Neighbor [76].

### Isolation by distance analysis

Stepping stone models on a two-dimensional space predict a linear relationship between Fst/(1-Fst) and the logarithm of geographic distance [77]. Because pairwise elements of distance matrices are not independent, a Mantel test with 10^4 ^permutations was used to test for the statistical significance of this relationship ['ecodist package' in R [78]]. In migration – drift equilibrium the variance of the genetic distance measure is further expected to increase with geographic distance [26]. We therefore assessed if the degree of scatter in the genetic distance measure increased with geographic distance. This was done by first obtaining the residuals from a standard linear regression of genetic distance (Fst/(1-Fst)) on the logarithm of geographic distance. These residuals and the log geographic distance matrix were then subjected to a partial Mantel test to test for statistical significance. As population structure can artificially produce statistically significant isolation by distance relationships, we also conducted partial Mantel tests correcting for the influence of habitat.

## Authors' contributions

JBWW conceived of the study, did the field work together with FT, conducted the genetic analyses and wrote the manuscript together with DT. CH was responsible for the stable isotope analysis, SB for the morphometric part of the study. SS helped to collect samples. DT hosted the project in his lab and together with FT provided significant input concerning the interpretation of the results. All authors read and approved of the final manuscript.

## Supplementary Material

Additional file 1 Condylobasal lengths for skulls from western and central habitats. Raw data of skull measurements Click here for file

Additional file 2 Membership coefficients of colonies to genetic clusters from STRUCTURE analysis. The data provided describes the proportions of individuals assigned to one of four population clusters given for each of the sampled rookeries. Clusters where the majority of individuals were assigned are highlighted. In addition, the mean of the greatest membership coefficients of each individual is reported for each of the sampled rookeries. Click here for file

Additional file 3 Specimens used for morphometrics. Specification of specimens used for morphometric analysis and the according Institutions that provided them. Click here for file

## References

[B1] Mallet J Speciation in the 21st century. Book review of "Speciation" by Jerry A. Coyne & H. Allen Orr Heredity 2005 95 105 109 10.1038/sj.hdy.6800686

[B2] Dieckmann U Doebeli M On the origin of species by sympatric speciation Nature 1999 400 6742 354 357 10.1038/22521 10432112

[B3] Bürger R Schneider KA Willensdorfer M The conditions for speciation through intraspecific competition Evolution 2006 60 2185 2206 17236413

[B4] Beltman JB Haccou P Speciation through the learning of habitat features Theor Popul Biol 2005 67 3 189 202 10.1016/j.tpb.2005.01.001 15808336

[B5] Doebeli M Dieckmann U Speciation along environmental gradients Nature 2003 421 259 264 10.1038/nature01274 12529641

[B6] Funk DJ Nosil P Etges WJ Ecological divergence exhibits consistently positive associations with reproductive isolation across disparate taxa Proc Natl Acad Sci USA 2006 103 9 3209 3213 10.1073/pnas.0508653103 16492742 PMC1413886

[B7] Dieckmann U Doebeli M Metz JAJ Tautz D Adaptive Speciation 2004 Cambridge, UK: Cambridge University Press

[B8] Rundle HD Nosil P Ecological speciation Ecol Lett 2005 8 3 336 352 10.1111/j.1461-0248.2004.00715.x

[B9] Schluter D The Ecology of Adaptive Radiation 2000 Oxford, U.K.: Oxford Universtiy Press

[B10] Steinfartz S Weitere M Tautz D Tracing the first steps to speciation: ecological and genetic differentiation of a salamader population in a small forest Mol Ecol 2007 16 21 4550 4561 10.1111/j.1365-294X.2007.03490.x 17877714

[B11] Tautz D Dieckmann U, Doebeli M, Metz JAJ, Tautz D Phylogeography and Patterns of Incipient Speciation Adaptive Speciation 2004 Cambridge: Cambridge University Press 305 320

[B12] Bekkevold D Andre C Dahlgren TG Clausen LAW Torstensen E Mosegaard H Carvalho GR Christensen TB Norlinder E Ruzzante DE Environmental correlates of population differentiation in Atlantic herring Evolution 2005 59 12 2656 2668 16526512

[B13] Hendry AP Wenburg JK Bentzen P Volk EC Quinn TP Rapid evolution of reproductive isolation in the wild: evidence from introduced salmon Science 2000 290 516 519 10.1126/science.290.5491.516 11039932

[B14] Tucker VA The energetic cost of moving about Am Scientist 1975 63 413 419 1137237

[B15] Fontaine MC Baird SJE Piry S Ray N Tolley KA Duke S Birkun A Ferreira M Jauniaux T Llavona A Rise of oceanographic barriers in continuous populations of a cetacean: the genetic structure of harbour porpoises in Old World waters BMC Biology 2007 5 10.1186/1741-7007-5-30 PMC1971045 17651495

[B16] Geyer LB Palumbi SR Reproductive character displacement and the genetics of gamete recognition in tropical sea urchins Evolution 2003 57 5 1049 1060 12836822 10.1111/j.0014-3820.2003.tb00315.x

[B17] Hoelzel AR Hey J Dahlheim ME Nicholson C Burkanov V Black N Evolution of population structure in a highly social top predator, the killer whale Mol Biol Evol 2007 24 6 1407 1415 10.1093/molbev/msm063 17400573

[B18] Wolf JB Tautz D Trillmich F Galápagos and Californian sea lions are separate species: genetic analysis of the genus Zalophus and its implications for conservation management Front Zool 2007 4 1 20 10.1186/1742-9994-4-20 17868473 PMC2072946

[B19] Banks S Danulat E, Edgar GJ Ambiente físico Reserva Marina de Galápagos 2002 Puerto Ayora, Santa Cruz, Galápagos: Fundación Charles Darwin/Servicio Parque Nacional Galápagos 22 35

[B20] Sakamoto CM Millero FJ Yao WS Friederich GE Chavez FP Surface seawater distributions of inorganic carbon and nutrients around the Galápagos Islands: results from the PlumEx experiment using automated chemical mapping Deep-Sea Res II 1998 45 6 1055 1071 10.1016/S0967-0645(98)00013-7

[B21] Vargas H Logheed C Snell H Population size and trends of the Galápagos Penguin *Spheniscus mendiculus* Ibis 2005 147 367 374 10.1111/j.1474-919x.2005.00412.x

[B22] Danulat E Edgar GJ Reserva Marina de Galápagos Puerto Ayora, Santa Cruz, Galápagos: Fundación Charles Darwin/Servicio Parque Nacional Galápagos; 2002

[B23] France RL Carbon-13 enrichment in benthic compared to planktonic algae: foodweb implications Mar Ecol – Prog Ser 1995 124 307 312 10.3354/meps124307

[B24] Hückstädt LA Rojas CP Antezana T Stable isotope analysis reveals pelagic foraging by the Southern sea lion in central Chile J Exp Mar Biol Ecol 2007 in press

[B25] Bearhop S Adams CE Waldron S Fuller RA Macleod H Determining trophic niche width: a novel approach using stable isotope analysis J Anim Ecol 2004 73 5 1007 1012 10.1111/j.0021-8790.2004.00861.x

[B26] Hutchison DW Templeton AR Correlation of pairwise genetic and geographic distance measures: Inferring the relative influences of gene flow and drift on the distribution of genetic variability Evolution 1999 53 6 1898 1914 10.2307/2640449 28565459

[B27] Weise MJ Costa DP Kudela RM Movement and diving behavior of male California sea lion (Zalophus californianus) during anomalous oceanographic conditions of 2005 compared to those of 2004 Geophys Res Lett 2006 33 22

[B28] Villegas-Amtmann S Costa DP Tremblay Y Aurioles-Gamboa D Salazar S Multiple foraging strategies in a marine apex predator, the Galápagos Sea Lion Mar Ecol – Prog Ser in press

[B29] Hoffman JI Matson CW Amos W Loughlin TR Bickham JW Deep genetic subdivision within a continuously distributed and highly vagile marine mammal, the Steller's sea lion (*Eumetopias jubatus*) Mol Ecol 2006 15 10 2821 2832 16911203 10.1111/j.1365-294X.2006.02991.x

[B30] Fabiani A Hoelzel AR Galimberti F Muelbert MMC Long-range paternal gene flow in southern elephant seals Science 2003 299 676 10.1126/science.299.5607.676 12560542

[B31] Grant PR Grant BR Evolution of Character Displacement in Darwin's Finches Science 2006 313 224 226 10.1126/science.1128374 16840700

[B32] Schluter D McPhail JD Ecological character displacement and speciation in sticklebacks Am Nat 1992 140 1 85 108 10.1086/285404 19426066

[B33] Trillmich F Ono KA Ecological Studies – Pinnipeds and El Niño 1991 88 Berlin: Springer Verlag

[B34] Wolf JBW Kauermann G Trillmich F Males in the shade: habitat use and sexual segregation in the Galápagos sea lion (*Zalophus californianus wollebaeki*) Behav Ecol Sociobiol 2005 59 2 293 302 10.1007/s00265-005-0042-7

[B35] Salazar SK Dieta, tamaño poblacional e interacción con desechos costeros del lobo marino Zalophus californianus wollebaeki en las islas Galápagos. Disertación previa al título de Licenciatura en Ciencias Biológicas Pontificia Universidad Católica del Ecuador 1999

[B36] Dellinger T Trillmich F Fish prey of the sympatric Galápagos fur seal and sea lions: seasonal variation and niche seperation Can J Zool 1999 77 1204 1216 10.1139/cjz-77-8-1204

[B37] Kooyman GL Trillmich F Gentry RL, Kooyman GL Diving Behavior of Galápagos Sea Lions Fur seals – Maternal Strategies on Land and at Sea 1986 Princeton, New Jersey: Princeton University Press 209 219

[B38] Stamps JA Davis JM Adaptive effects of natal experience on habitat selection by dispersers Anim Behav 2006 72 1279 1289 10.1016/j.anbehav.2006.03.010

[B39] Slagsvold T Wiebe KL Learning the ecological niche Proc R Soc B 2007 274 1606 19 23 10.1098/rspb.2006.3663 PMC1679873 17015332

[B40] Musiani M Leonard JA Cluff HD Gates CC Mariani S Paquet PC Vilà C Wayne RK Differentiation of tundra/taiga and boreal coniferous forest wolves: genetics, coat colour and association with migratory caribou Mol Ecol 2007 16 4149 4170 10.1111/j.1365-294X.2007.03458.x 17725575

[B41] Trillmich F Wolf JBW Parent-offspring and sibling conflict in Galápagos fur seals and sea lions Behav Ecol Sociobiol 2008 62 3 363 375 10.1007/s00265-007-0423-1

[B42] Kastak CR Schusterman RJ Long-term memory for concepts in a California sea lion (*Zalophus californianus*) Anim Cogn 2002 5 4 225 232 10.1007/s10071-002-0153-8 12461600

[B43] Schusterman RJ Reichmuth CJ Kastak D How animals classify friends and foes Curr Dir Psychol Sci 2000 9 1 1 6 10.1111/1467-8721.00047

[B44] Wolf JBW Trillmich F Beyond habitat requirements: individual fine-scale site fidelity in a colony of the Galapagos sea lion (*Zalophus wollebaeki*) creates conditions for social structuring Oecologia 2007 152 553 567 10.1007/s00442-007-0665-7 17505851

[B45] Wolf JBW Mawdsley D Trillmich F James R Social structure in a colonial mammal: Unravelling hidden structural layers and their foundations by network analysis Anim Behav 2007 74 5 1293 1302 10.1016/j.anbehav.2007.02.024

[B46] Wolf JBW Trillmich F Kin in space. Social viscosity in a spatially and genetically sub-structured network Proc R Soc Lond B 2008 10.1098/rspb.2008.0356 PMC2603206 18522913

[B47] Campbell RA Gales NJ Lento GM Baker CS Islands in the sea: extreme female natal site fidelity in the Australian sea lion, *Neophoca cinerea* Biol Lett 2008 4 139 142 10.1098/rsbl.2007.0487 18042512 PMC2412930

[B48] Greenwood PJ Mating systems, philopatry and dispersal in birds and mammals Anim Behav 1980 28 1140 1162 10.1016/S0003-3472(80)80103-5

[B49] Voigt BD Social organization and territoral behavior of the Galápagos sea lion *Zalophus californianus wollebaeki *(Sivertsen, 1953) Thesis. Kopenhagen 1979 1 75

[B50] East ML Burke T Wilhelm K Greig C Hofer H Sexual conflicts in spotted hyenas: male and female mating tactics and their reproductive outcome with respect to age, social status and tenure Proc Biol Sci 2003 270 1521 1247 1254 10.1098/rspb.2003.2363 12816637 PMC1691369

[B51] Poston JP Dominance, access to colonies, and queues for mating opportunities by male boat-tailed grackles Behav Ecol Sociobiol 1997 41 2 89 98 10.1007/s002650050368

[B52] Kunc H Wolf JBW Seasonal changes of vocal rates and their relation to territorial status in male Galápagos sea lions (*Zalophus wollebaeki*) Ethology 2008 114 381 388 10.1111/j.1439-0310.2008.01484.x

[B53] Hoffman JI Trathan PN Amos W Genetic tagging reveals extreme site fidelity in territorial male Antarctic fur seals Arctocephalus gazella Mol Ecol 2006 15 12 3841 3847 10.1111/j.1365-294X.2006.03053.x 17032279

[B54] Hoffman JI Boyd IL Amos W Male reproductive strategy and the importance of maternal status in the antarctic fur seal *Arctocephalus gazella* Evolution 2003 57 8 1917 1930 14503632 10.1111/j.0014-3820.2003.tb00598.x

[B55] Hendry AP Selection against migrants contributes to the rapid evolution of ecologically dependent reproductive isolation Evol Ecol Res 2004 6 1219 1236

[B56] Nosil P Vines TH Funk DJ Reproductive isolation caused by natural selection against immigrants from divergent habitats Evolution 2005 59 705 719 15926683

[B57] Simkin T Perry R Geology of Galápagos Islands Key environments – Galápagos 1984 Oxford: Pergamon Press 15 43

[B58] Kurz MD Rowland S Curtice J Saal A Naumann T Eruption rates at Fernandina volcano, Galápagos archipelago, from cosmogenic helium surficial laval flows Eos Trans AGU 2005 86 52 Fall Meet. Suppl., Abstract U33A-0016

[B59] Naumann T Geist D Physical volcanology and structural development of Cerro Azul volcano, Isabela island, Galápagos: implications for the development of Galápagos-type shield volcanoes Bull Volcanol 2000 61 497 514

[B60] Harrod C Grey J Isotopic variation complicates analysis of trophic relations within the fish community of Plußsee: a small, deep, stratifying lake Archiv für Hydrobiologie 2006 167 281 299 10.1127/0003-9136/2006/0167-0281

[B61] Harrod C Grey J McCarthy TK Morrissey M Stable isotope analyses provide new insights into ecological plasticity in a mixohaline population of European eel Oecologia 2005 144 673 683 10.1007/s00442-005-0161-x 16025352

[B62] Brunner S Bryden MM Shaughnessy PD Cranial ontogeny of otariid seals Syst Biodivers 2004 2 1 83 110 10.1017/S1477200004001367

[B63] EMBL Align database http://www.ebi.ac.uk/embl/Submission/alignment.html OR http://srs.ebi.ac.uk OR ftp://ftp.ebi.ac.uk/pub/databases/embl/align/

[B64] Excoffier L Laval G Schneider S Arlequin ver. 3.0: An integrated software package for population genetics data analysis Evol Bioinformatics Online 2005 1 47 50 PMC2658868 19325852

[B65] Rassmann K Tautz D Trillmich F Gliddon C The microevolution of the Galápagos marine iguana *Amblyrhynchus cristatus *assessed by nuclear and mitochondrial genetic analyses Mol Ecol 1997 6 5 437 452 10.1046/j.1365-294X.1997.00209.x

[B66] Wolf JBW Tautz D Caccone A Steinfartz S Development of new microsatellite loci and evaluation of loci from other pinniped species for the Galápagos sea lion (*Zalophus californianus wollebaeki*) Conserv Genet 2006 7 3 461 465 10.1007/s10592-005-9045-1

[B67] Hoffman JI Steinfartz S Wolf JBW Ten novel dinucleotide microsatellite loci cloned from the Galápagos sea lion (*Zalophus californianus wollebaeki*) are polymorphic in other pinniped species Mol Ecol Notes 2007 7 1 103 105 10.1111/j.1471-8286.2006.01544.x

[B68] Falush D Stephens M Pritchard JK Inference of population structure using multilocus genotype data: Linked loci and correlated allele frequencies Genetics 2003 164 4 1567 1587 12930761 10.1093/genetics/164.4.1567 PMC1462648

[B69] Evanno G Regnaut S Goudet J Detecting the number of clusters of individuals using the software STRUCTURE: a simulation study Mol Ecol 2005 14 2611 2620 10.1111/j.1365-294X.2005.02553.x 15969739

[B70] Pritchard JK Stephens M Donnelly P Inference of population structure using multilocus genotype data Genetics 2000 155 2 945 959 10835412 10.1093/genetics/155.2.945 PMC1461096

[B71] Weir BS Cockerham CC Estimating F-statistics for the analysis of population structure Evolution 1984 38 1358 1370 10.2307/2408641 28563791

[B72] Goodman SJ R-ST Calc: a collection of computer programs for calculating estimates of genetic differentiation from microsatellite data and determining their significance Mol Ecol 1997 6 9 881 885 10.1111/j.1365-294X.1997.tb00143.x

[B73] Goudet J FSTAT: a program to estimate and test gene diversities and fixation indices (version 2.9.3) 2001 http://www.unil.ch/izea/softwares/fstat.html

[B74] Goudet J Raymond M De Meeüs T Rousset F Testing differentiation in diploid populations Genetics 1996 144 1933 1949 8978076 10.1093/genetics/144.4.1933 PMC1207740

[B75] Minch E Ruiz-Linares A Goldstein DB Feldman M Cavalli-Sforza LL Microsat (version 1.5d): a program for calculating various statistics on microsatellite allele data 1997 Stanford, CA: Stanford University

[B76] Felsenstein J PHYLIP (Phylogeny Inference Package) version 3.6 Distributed by the author Department of Genome Sciences, University of Washington, Seattle 2004

[B77] Rousset F Genetic differentiation between individuals J Evol Biol 2000 13 1 58 62 10.1046/j.1420-9101.2000.00137.x

[B78] Ihaka R Gentleman R R: a language for data analysis and graphics J Comput Graph Stat 1996 5 299 314 10.2307/1390807

