# Supplementary material for: Tracing early stages of species differentiation: Ecological, morphological and genetic divergence of Galápagos sea lion populations
Source: BMC Evol Biol. 2008 May 16;8:150. doi: 10.1186/1471-2148-8-150 (PMC2408593; doi:10.1186/1471-2148-8-150)
Supplement: Additional file 1 — Condylobasal lengths for skulls from western and central habitats. Raw data of skull measurements [file 1471-2148-8-150-S1.doc]

**Additional file 1:** Condylobasal lengths for skulls from western and central habitats

| Sex | West | | | Centre | | |
| --- | --- | --- | --- | --- | --- | --- |
|  | *n* | Mean | St. Dev | *n* | Mean | St. Dev |
| Males | 5 | 257.91 | 3.81 | 19 | 263.16 | 6.74 |
| Females | 4 | 220.49 | 1.98 | 15 | 225.19 | 4.71 |
