# Supplementary material for: Tracing early stages of species differentiation: Ecological, morphological and genetic divergence of Galápagos sea lion populations
Source: BMC Evol Biol. 2008 May 16;8:150. doi: 10.1186/1471-2148-8-150 (PMC2408593; doi:10.1186/1471-2148-8-150)
Supplement: Additional file 2 — Membership coefficients of colonies to genetic clusters from STRUCTURE analysis. The data provided describes the proportions of individuals assigned to one of four population clusters given for each of the sampled rookeries. Clusters where the majority of individuals were assigned are highlighted. In addition, the mean of the greatest membership coefficients of each individual is reported for each of the sampled rookeries. [file 1471-2148-8-150-S2.doc]

**Additional file 2:** Proportions of individuals assigned to one of four population clusters given for each of the sampled rookeries. Clusters where the majority of individuals were assigned are highlighted. In addition, the mean of the greatest membership coefficients of each individual is reported for each of the sampled rookeries.

| Population |  | Population cluster | | | | Membership coefficient [mean±SE] |  |
| --- | --- | --- | --- | --- | --- | --- | --- |
|  |  | 1 | 2 | 3 | 4 | mean  se | sample size |
| CA | Centre | 0 | **0.50** | 0.40 | 0.10 | 0.679 ± 0.024 | 30 |
| ILZN |  | 0 | **0.53** | 0.30 | 0.17 | 0.717 ± 0.022 | 47 |
| CF |  | 0 | **0.41** | 0.31 | 0.28 | 0.685 ± 0.030 | 29 |
| SF |  | 0 | **0.56** | 0.28 | 0.15 | 0.715 ± 0.021 | 39 |
| PC |  | 0 | **0.40** | 0.27 | 0.33 | 0.712 ± 0.033 | 30 |
| ECEG |  | 0 | 0.32 | **0.50** | 0.18 | 0.750 ± 0.025 | 28 |
| MO |  | 0 | 0.30 | **0.47** | 0.23 | 0.685 ± 0.027 | 40 |
| SA |  | 0 | 0.30 | **0.47** | 0.23 | 0.680 ± 0.031 | 30 |
| GE |  | 0 | 0.14 | **0.57** | 0.29 | 0.706 ± 0.032 | 14 |
| IV |  | 0 | **0.33** | **0.33** | **0.33** | 0.725 ± 0.033 | 30 |
| IBES | West | 0 | 0.07 | 0.19 | **0.74** | 0.761 ± 0.042 | 27 |
| FH |  | 0 | 0 | 0.04 | **0.96** | 0.862 ± 0.035 | 23 |
| *Zalophus californianus* | Outgroup | **1.00** | 0 | 0 | 0 | 1.000 ± 0 | 16 |
