# Supplementary material for: Tracing early stages of species differentiation: Ecological, morphological and genetic divergence of Galápagos sea lion populations
Source: BMC Evol Biol. 2008 May 16;8:150. doi: 10.1186/1471-2148-8-150 (PMC2408593; doi:10.1186/1471-2148-8-150)
Supplement: Additional file 3 — Specimens used for morphometrics. Specification of specimens used for morphometric analysis and the according Institutions that provided them. [file 1471-2148-8-150-S3.doc]

**Additional file 3:** Specimens used for morphometrics

**AMNH** – American Museum of Natural History (New York, United States): 63946, 99461, 99462, 99463, 214780, 214781. **FMNH** – Field Museum of Natural History (Chicago, United States): 51748, 51760. **MNHN -** Muséum national d'Histoire naturelle (Paris, France): 1962.1142, 1973.293. **NMNH** – National Museum of Natural History (Washington DC, United States): 23276, 23277, 23278, 23279, 23280, 23281, 396917. **SDNHM** – San Diego Natural History Museum (San Diego, United States): 22869. **ZFMK** - Zoologisches Forschungsinstitut und Museum Alexander Koenig (Bonn, Germany): 2005_478, 2005_510, 2005_512, 2005_516, 2005_532, 2005_481, 2005_536, 2005_566, 2005_574, 2005_520, 2005_537, 2005_584, 2005_484, 2005_542, 2005_556, 2005_560, 2005_598, 2005_563, 2005_564, 2005_492, 2005_494, 2005_495, 2005_500, 2005_482, 2005_470
